# HOXB4 promotes the malignant progression of ovarian cancer via DHDDS

**DOI:** 10.1186/s12885-020-06725-4

**Published:** 2020-03-16

**Authors:** Na Li, Jin-hai Gou, Jiao Xiong, Juan-juan You, Zheng-yu Li

**Affiliations:** 1 Department of Gynecology and Obstetrics, West China Second University Hospital, Sichuan University, Chengdu, 610041 People’s Republic of China; 2 Key Laboratory of Obstetrics & Gynecologic and Pediatric Diseases and Birth Defects of Ministry of Education, West China Second University Hospital, Sichuan University, Chengdu, Sichuan 610041 P.R. China; 3 Department of Obstetrics and Gynecology, The first affiliated Hospital of Zunyi Medical University, Zunyi, Guizhou 563000 People’s Republic of China

**Keywords:** Ovarian cancer, Homeobox B4, Malignant progression, DHDDS

## Abstract

**Background:**

Homeobox B4 (HOXB4) is correlated with poor prognosis of various cancer types. However, how HOXB4 promotes ovarian cancer (OV) progression remains unclear.

**Methods:**

The Cancer Genome Atlas (TCGA) database indicated that a high level of HOXB4 in OV was correlated with poor prognosis. The biological functions of HOXB4 were confirmed by colony formation, migration, and invasion assays. The effect of HOXB4 on the expression of EMT cell markers was determined. The transcriptional target of HOXB4 was DHDDS, which was detected by a ChIP assay. A xenograft tumor model was generated in nude mice to detect the role of HOXB4 in tumor proliferation and metastasis.

**Results:**

The results showed that HOXB4 protein levels were higher in OV tissues than in normal tissues and correlated with poor prognosis of OV. HOXB4 reduction inhibited the proliferation and invasion ability of OV cells in vitro. Conversely, these effects were enhanced by the upregulation of HOXB4 in OV cells. The binding of HOXB4 to two DNA motifs regulated DHDDS expression and contributed to the malignant progression of OV. The role of HOXB4 in contributing to tumor development in vivo was verified in mice. Further results indicated that HOXB4 induced Snail and Zeb1 expression.

**Conclusion:**

Overall, HOXB4 overexpression was remarkably correlated with poor prognosis of OV. Mechanistically, HOXB4 enhances the proliferation and invasion of tumor cells by activating DHDDS, thereby promoting the malignant progression of OV.

## Background

Ovarian cancer (OV) is predicted to be the second-deadliest type of cancer among women. Among gynecologic cancers OV has a poor prognosis [1–4]. Although many target drugs have been applied to OV therapy, the death rate of OV patients is still increasing every year [5–8]. OV shows resistance to chemotherapy, radiotherapy, and molecularly targeted therapy [9]. Complex epigenetic changes pose a major challenge to OV therapy. Epithelial–mesenchymal transition (EMT) is the main malignant progression mechanism driving tumor cell metastasis and invasion [10–13]. Multiple signaling pathways and transcriptional factors, such as Snail, Zeb1, Twist, and Slug, are involved in the regulation of EMT [12–17]. New transcriptional factors that drive EMT should be discovered to indicate OV malignant progression.

The homeobox (HOXB) family is crucial for cell morphogenesis and differentiation [18–20]. The HOXB family involvement in tumor EMT has yet to be fully investigated, and EMT-associated HOXBs in OV have rarely been reported [18–22]. Homeobox B4 (HOXB4) is an important transcription factor involved in the progression of lung, breast, prostate, and bladder cancer [23–27]. HOXB4 enhances proliferation and the stat3 pathway [28–32]. HOXB4 weakens the cytotoxic effect of paclitaxel and cisplatin by downregulating ABC transporters in OV [33]. Although HOXB4 overexpression is significantly correlated with cancer progression and poor prognosis, the precise mechanism of HOXB4 in OV remains unclear.

In this study, we revealed the potential mechanism of HOXB4 in OV malignant progression. First, we found that the overexpression of HOXB4 in OV tissues is closely correlated with a short survival rate in patients with OV. We also investigated whether HOXB4 can promote cell proliferation, invasion, and migration of OV in vitro and in vivo. Overall, this study explains the mechanism by which HOXB4 regulates the malignant progression of OV.

## Methods

### Clinical samples and TCGA database analysis

TCGA (The Cancer Genome Atlas) datasets for OV were used to analyze gene expression and survival rate. Twenty fresh samples with adjacent normal tissues were obtained from surgical cases. Fresh tissues were used to detect the expression of HOXB4 in OV. All patients were informed.

### Gene annotation and enrichment analysis

We used Metascape (metascape.org) for gene enrichment analysis of HOXB4. Metascape is an online bioinformatics pipeline with multiple gene lists that supports effective data-driven gene prioritization decisions.

### Cell culture

The human OV cell lines SKOV3 and OVCAR3 were obtained from Shanghai Institute of Cell Biology (Cat. TCHu185 and TCHu228, Shanghai, China) at 2019. All cells have been identified by STR before purchase. During the experiment, we have performed a Mycoplasma test every 2 months and confirm that the cells are not contaminated. Cells were maintained in DMEM (dulbecco’s modified eagle medium) (Gibco, USA) with 10% FBS (fetal bovine serum) (Gibco, USA) at 37 °C and 5% CO_2_.

### RNA interference

shRNAs targeting HOXB4 were purchased from Origene Biotechnology Company (Beijing, China). The interference efficiency of shRNAs was detected by Western blot after transfection for 48 h.

### Colony formation assay

A colony formation assay was performed to analyze cell proliferation. Cells were seeded in a six-well plate at a final concentration of 100 cells/well. After culturing for 15 days, the cells were fixed and stained with 0.5% crystal violet (Sigma, USA). Colonies with more than 50 cells were imaged and counted.

### Invasion assay

Transwell inserts with (8 μm pore size, Millipore, USA) were used to detect cell invasion ability. Cells were added to the upper insert chamber and cultured with serum-free DMEM, and the lower culture chamber was filled with DMEM containing 20% FBS. Thirty-six hours later, after the cells in the upper chamber were removed, the remaining invading cells were fixed and stained with crystal violet. The number of cells was counted under a light microscope (Nikon, Japan).

### Migration assay

OVCAR3 and SKOV3 cells were seeded in 24-well plates and cultured for 24 h. A linear wound was created, and the cells were washed with PBS 3 times. Then, complete medium was added and cultured for 36 h. Finally, images were taken at 0, 18, and 36 h, and the scratched area was recorded.

### Western blot analysis

Total proteins harvested from cells and tumor samples were separated by sodium dodecyl sulfate–polyacrylamide gel electrophoresis (SDS-PAGE) and transferred to PVDF (Polyvinylidene Fluoride) membranes. Then, the membranes were blocked with 5% skimmed milk and incubated with the following specific primary antibodies at 4 °C overnight: anti-HOXB4, anti-E-cadherin, anti-Vimentin, anti-Snai1, and anti-Zeb1 antibody. GAPDH was used as a loading control. After washing with PBST, the membranes were incubated with HRP-labeled secondary antibodies (Sigma, USA). Protein intensity was detected by Image Lab (Bio-Rad, USA).

### ChIP-seq data analysis

The ChIP-seq data were downloaded from Cistrome Data (http://dc2.cistrome.org/#/). To verify the genes that HOXB4 binds to and their corresponding motifs, we used ChIPseeker to analyze the downloaded data according to the R package and method provided by YuLab [34].

### Luciferase reporter assay

The DHDDS motifs were amplified from human genomic DNA and cloned into a pGL4.3 luciferase reporter vector (Promega). Transactivation assays were performed using the Dual-Luciferase Reporter Assay System (Promega). Luciferase activities were measured using a Synergy 2 microplate reader system (Gene).

### Zymography assays

All media were collected and subjected to SDS-PAGE with 0.01% wt/vol gelatin. After electrophoresis, gels were stained with Coomassie R250 and destained until the wash became clear with apparent cleared zones associated with MMP (matrix metallopeptidase 2) activity.

### Xenograft model

To verify whether the effect of HOXB4 in animals is consistent with the results of in vitro experiments, a total of 18 6-week-old BALB/c nude mice were purchased from Vital River (Beijing, China) and randomly divided into 4 groups: OVCAR3/nc, OVCAR3/HOXB4 (OVCAR3 cells stably expressing HOXB4), OVCAR3/DHDDS (OVCAR3 cells stably expressing DHDDS) and OVCAR3/HOXB4 + siDHDDS (mice stably expressing HOXB4 were treated with DHDDS siRNA after tumor formation). A total of 1 × 10^6^ cells were injected subcutaneously or in the tail vein. All animals were euthanized by intravenous injection of barbiturate at a final concentration of 100 mg/kg, and then the tumors were removed and fixed in paraffin for further analysis. The tumor volume was calculated as follows: tumor volume = length × width^2^/2. All procedures involving animals were in accordance with the ethical standards of the Institutional Animal Care and Use Committee (IACUC) at West China Second University Hospital.

### Histology and immunohistochemistry (IHC)

Tumor tissue from nude mice was embedded and cut into 4 μm-thick sections. After microwave oven/3% H_2_O_2_ treatment, the following primary antibodies were added: anti-HOXB4 antibody (1:500; Abcam, UK), anti-MMP2 antibody (1:500; Abcam, UK), anti-MMP9 antibody (1:300; Abcam, UK), anti-E-cadherin antibody (1:500; Abcam, UK), and anti-vimentin antibody (1500; Abcam, UK) at 4 °C overnight. The immunohistochemical staining results were collected and scored by professionals.

### Statistical analysis

Statistical analyses were performed using SPSS 21.0 (SPSS Inc., USA). Statistically significant differences were analyzed using Student’s t-test and one-way ANOVA. Differences were considered significant at *P* < 0.05 and labeled with *.

## Results

### A high level of HOXB4 is correlated with poor prognosis in OV

**Fig. 1:**
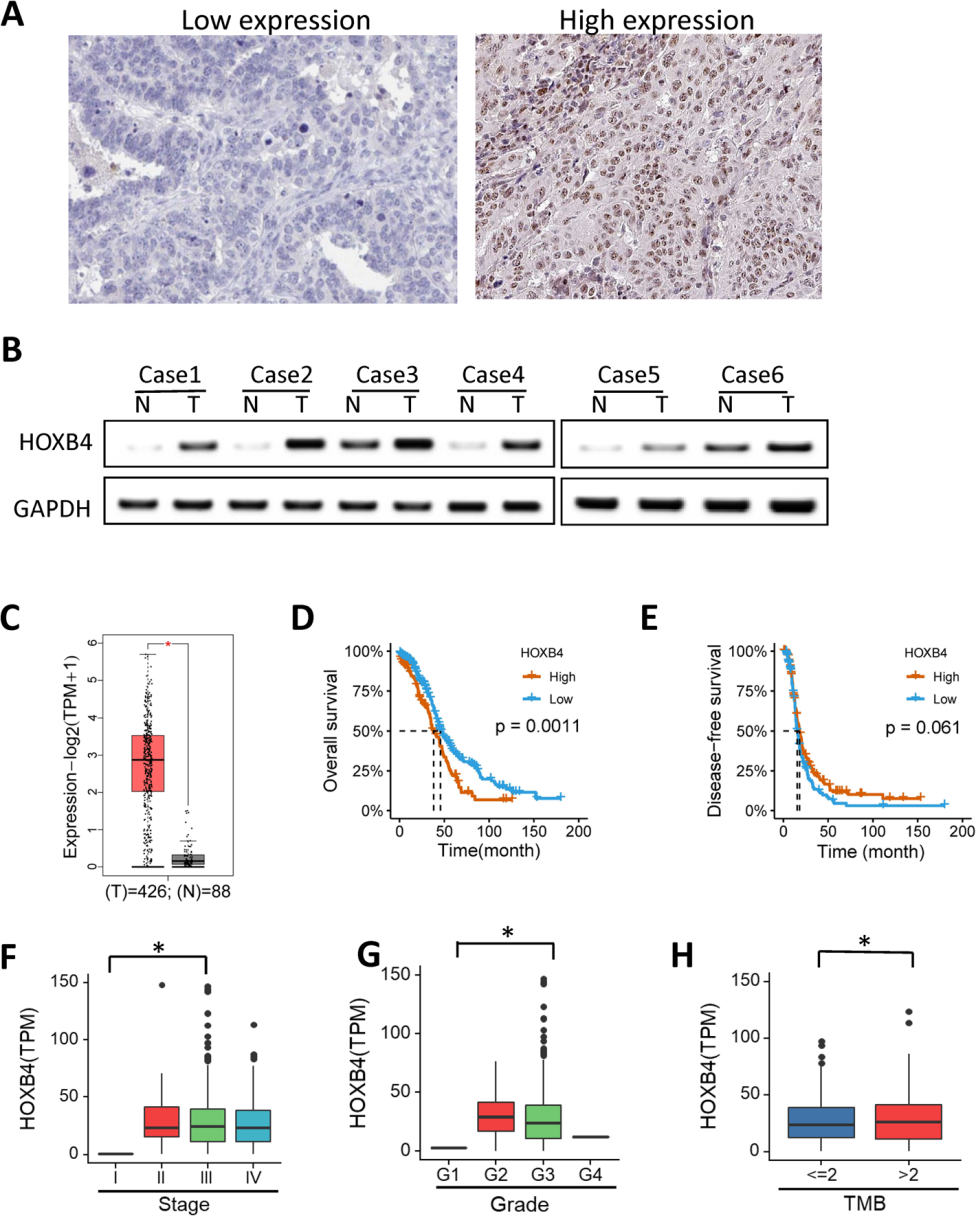
HOXB4 upregulation in OV tissues was correlated with poor prognosis. **a** IHC staining showed high and low HOXB4 expression levels in OV tissues. **b** Representative WB revealed the expression level of HOXB4 in OV and paired adjacent noncancerous tissues. **c** HOXB4 expression was negative in adjacent noncancerous tissue and positive in OV tissue. **d** Kaplan–Meier analysis of OS in patients with OV. A high level of HOXB4 was associated with a short survival time in patients with OV; a low level of HOXB4 was correlated with a long survival time (*P* < 0.05). **e** PFS in OV showed results similar to OS. **f** HOXB4 was positive in high grades of OV (P < 0.05). **g** HOXB4 was positively correlated with tumor mutational burden (TMB; P < 0.05). **h** HOXB4 was positively correlated with lymph node metastasis (P < 0.05). Statistically significant differences of were analyzed using Student’s t-test

The expression of HOXB4 in 373 cases of OV specimens from TCGA and 6 cases of fresh OV tissue with normal tissues was detected to examine the correlation between HOXB4 and OV prognosis. The expression level of HOXB4 was higher in OV tissues than in normal tissues. IHC results revealed the high expression level of HOXB4 in OV tumor tissues (Fig. 1a). Western blot results showed that the protein level of HOXB4 was upregulated in randomly selected paired tumor specimens (Fig. 1b). In the TCGA database analysis, the TPM (tumor mutation burden) from RNA-seq revealed the variable expression of HOXB4 in OV tissues (Fig. 1c). The TCGA database analysis suggested that a high expression level of HOXB4 in OV was associated with short overall survival time (Fig. 1d) and PFS (Fig. 1e). We also found that the high expression of HOXB4 in OV tissue was positively associated with clinical stage (Fig. 1f), pathologic grade (Fig. 1g), and TMB (tumor mutation burden) of OV (Fig. 1h). Our data verified that the aberrant overexpression of HOXB4 in OV tissues was correlated with poor prognosis of OV.

### HOXB4 is a driver of malignant progression in the TCGA dataset

**Fig. 2:**
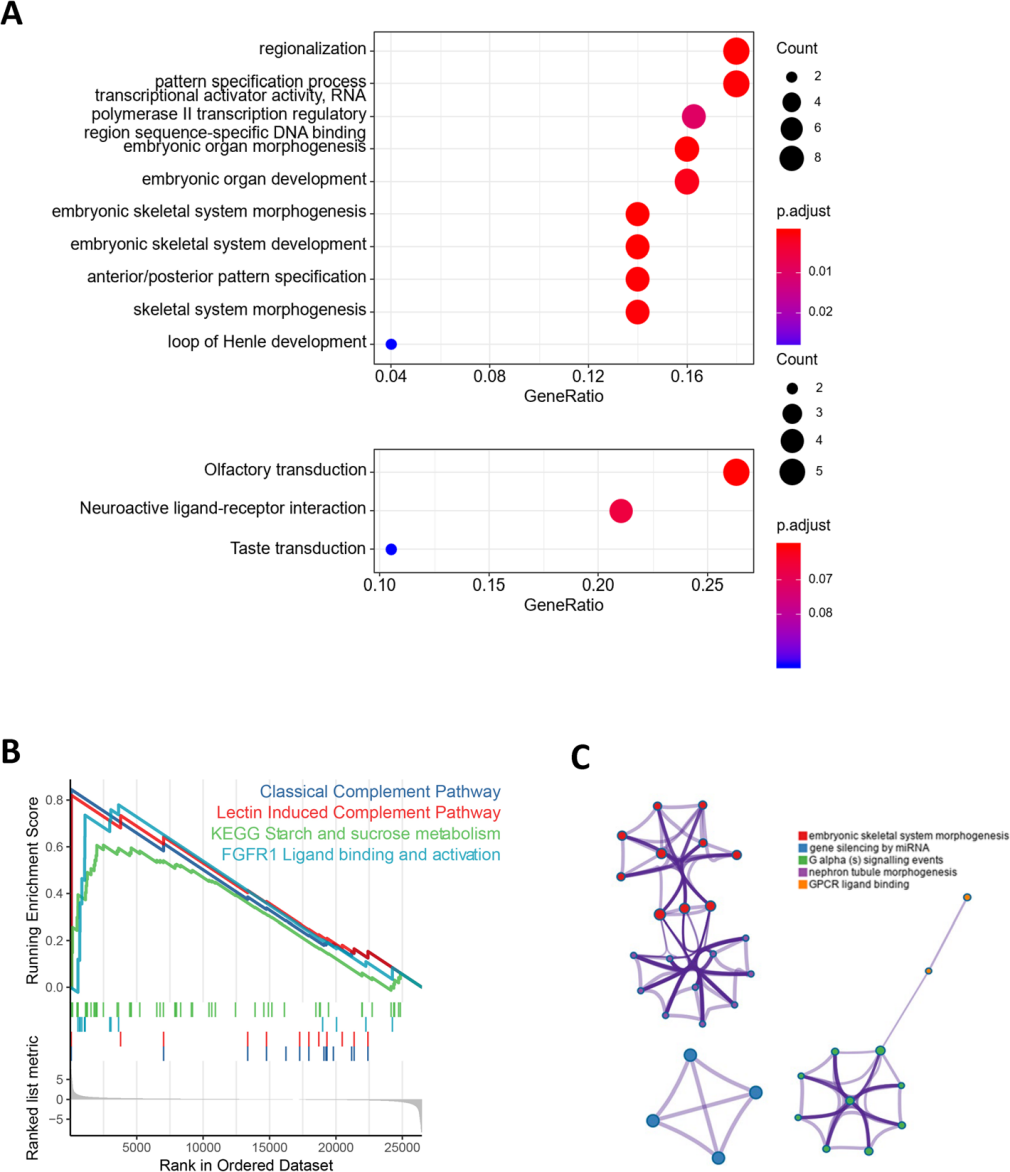
Bioinformatics analysis of patients with HOXB4 in TCGA with high expression and patients and low expression showed the differences between the spectra. **a** GO analysis showed that pathways associated with embryo morphogenesis were enriched. **b** GSEA revealed that multicancer invasiveness and embryonic stem cell core were enriched. **c** Metascape analysis also showed enrichment of embryonic-associated pathways

Based on the TCGA database, TPM data was extracted from each RNA-seq dataset to analyze the HOXB4 high-expression group compared with the low-expression group. The GO enrichment of differentially expressed genes revealed that embryonic development-related pathways were effectively enriched (Fig. 2a and b). GSEA (Gene Set Enrichment Analysis) showed that the embryonic development-associated pathway was also enriched between the HOXB4 high- and low-expression groups, as well as the epithelial cell differentiation pathway, signal transduction of gene expression pathway and others (Fig. 2c). In addition, the results of the Metascape analysis also suggested that HOXB4 was closely related to embryo morphogenesis and cell surface receptors (Fig. 2d). These data indicated that HOXB4 participated in cell differentiation and might play a driving role in OV progression.

### HOXB4 increases OV cell malignant progression in vitro

**Fig. 3:**
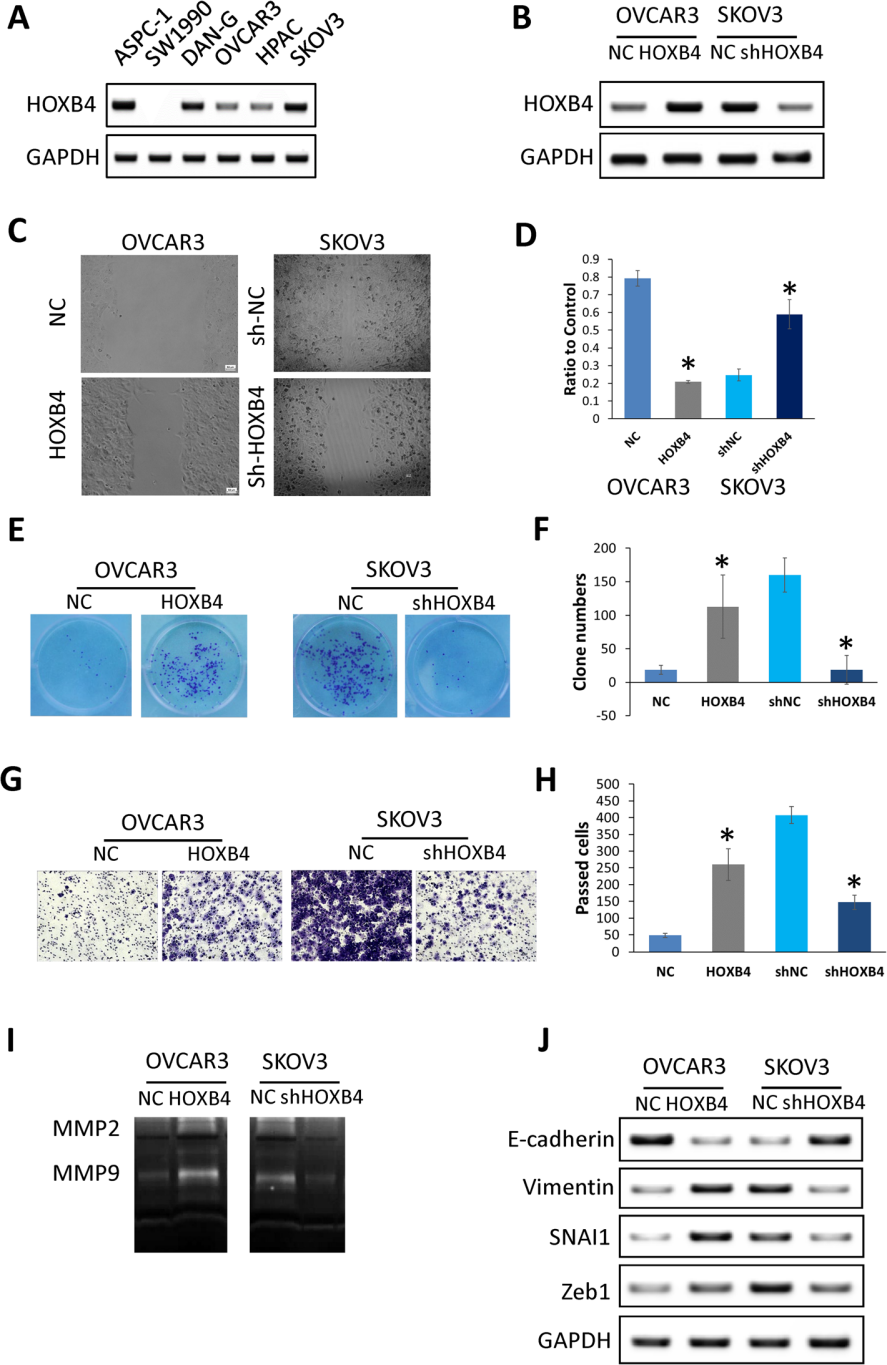
HOXB4 facilitated OV cell proliferation by promoting progression. **a** Protein level of HOXB4 in different OV lines. **b** Efficiency of HOXB4 knockdown and overexpression in SKOV3 and OVCAR3 cells, respectively. **c** & **d** Wound healing assay to detect the effect of HOXB4 in OVCAR3 and SKOV3 cells (P < 0.05, respectively). **e** & **f** A cell colony formation assay was used to examine the effect of HOXB4 on the proliferation of OV cells. HOXB4 promoted the proliferation of OV cells (P < 0.05). **g** & **h** Analysis of invasion by Transwell assay in OVCAR3 and SKOV3 cells. HOXB4 accelerated the invasion of OV cells (P < 0.05). **i** & **j** Zymography assays (**i**) and Western blot (**j**) to detect invasion and EMT marker expression levels after HOXB4 knockdown or overexpression. Statistically significant differences were analyzed using Student’s t-test

To further characterize the expression level of HOXB4, the protein level of HOXB4 expression in various OV cell lines was detected by Western blot analysis. The HOXB4 expression level in OVCAR3 cells was decreased, but it was higher in SKOV3 cells (Fig. 3a). OVCAR3 cells were transfected with OVDNA3.1-HOXB4. For SKOV3 cells, shRNA was used to knock down HOXB4 expression, as demonstrated by Western blot analysis (Fig. 3b). When HOXB4 expression was upregulated, the speed of wound healing was faster than that of the control group (Fig. 3c and d). Quantitative analysis of the colony formation results suggested a significant difference between cells with high and low HOXB4 expression (Fig. 3e and f). In the invasion assay, high expression of HOXB4 promoted the invasive capacity of ovarian cancer cells (Fig. 3g and h). MMPs are effector molecules that are critical in cell plasticity and EMT. Zymographic assays showed that the activities of MMP2 and MMP9 were significantly higher in the HOXB4-upregulated OVCAR3 cells than in the control group. The activity of MMP9 with HOXB4 in OVCAR3 cells was higher than that in the control group (Fig. 3i). For EMT markers, a high HOXB4 level was accompanied by upregulated vimentin, Snail, and Zeb1 and downregulated E-cadherin in OVCAR3 and SKOV3 cells (Fig. 3j).

### HOXB4 promotes DHDDS by binding to conserved motifs in the promoter region

**Fig. 4:**
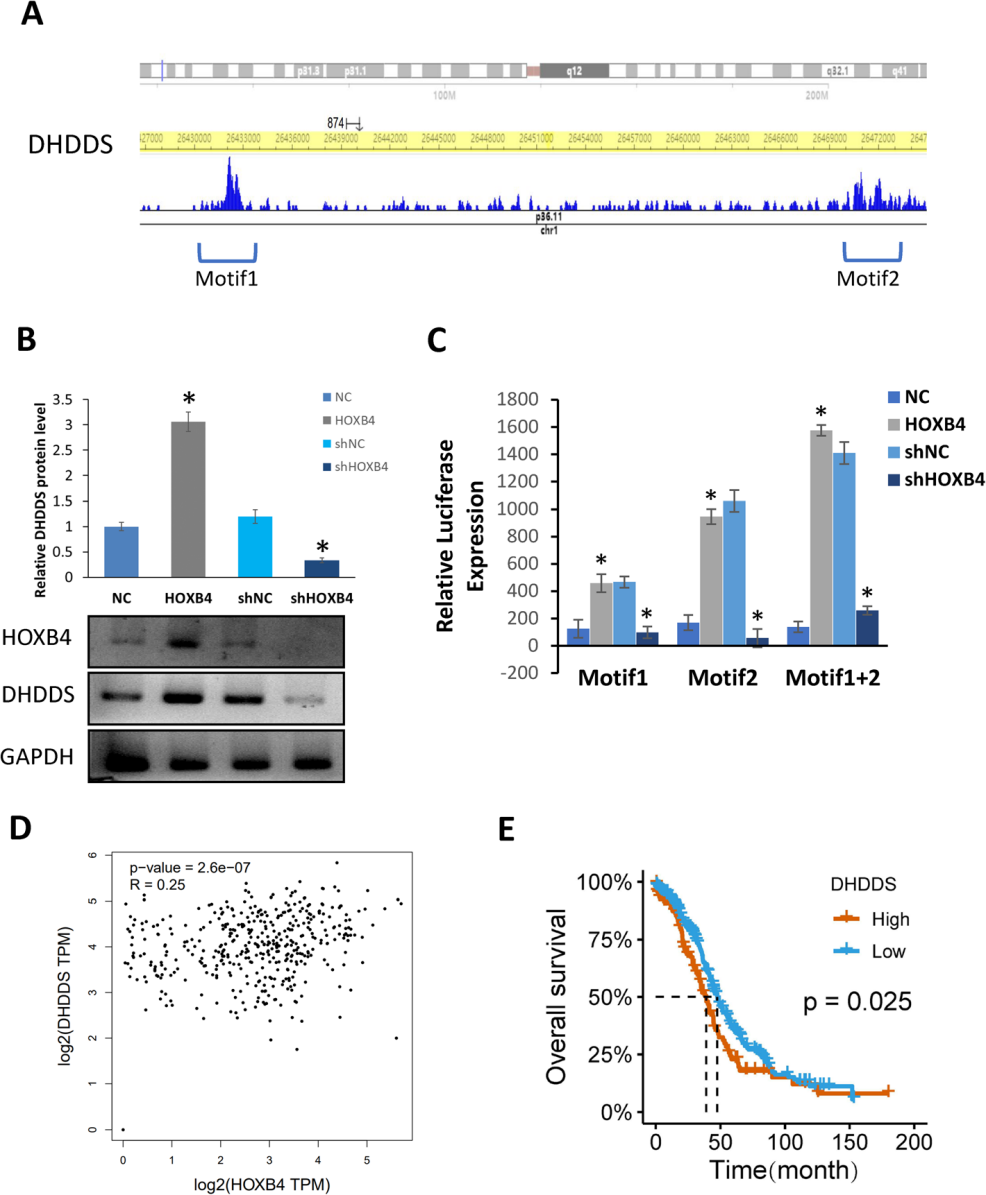
Motifs recognized by HOXB4 in its target DHDDS. **a** Motif analysis of the promoter region of the target gene DHDDS by using ChIPseeker. Two individual motifs and their locations within the promoter of DHDDS, Chr1 629,791–630,040 and Chr1 633,904–634,132, respectively. **b** Western blot was employed to detect the level of DHDDS after HOXB4 knockdown and overexpression; HOXB4 promoted DHDDS expression (P < 0.05). **c** Luciferase reporter assay showed that HOXB4 transcriptionally activated motifs 1 and 2, and the effect was enhanced by the 1 + 2 sequences together (P < 0.05). **d** TCGA analysis showed a correlation between HOXB4 and DHDDS expression in the OV samples. **e** The correlation between HOXB4 and OV patient survival time. Statistically significant differences were analyzed using Student’s t-test

On the basis of ChIP-seq in the Cistrome Data (http://dc2.cistrome.org/#/), we referred to Yan J.’s data published in Cell, 2013, in which HOXB4 was studied by ChIP analysis in HeLa cells. We screened the binding motif of HOXB4 in DHDDS. We defined motifs based on the Chip-seq peaks in the DHDDS promoter region by using ChIPseeker online software and motif analysis. Two individual motifs and their locations within the promoter of DHDDS were found. Motif 1 and motif 2 possessed the relatively conserved sequences of Chr1 629,791–630,040 and Chr1 633,904–634,132 (Fig. 4a). Then, we upregulated the expression of HOXB4 in OVCAR3 cells and knocked down HOXB4 in SKOV3 cells to detect DHDDS expression levels. The results showed that DHDDS expression was upregulated following HOXB4 overexpression (Fig. 4b). Truncated luciferase reporter plasmids were used for motif characterization. The DHDDS motifs were truncated into two regions, one of which covered its peak sequence. The luciferase assay data showed that HOXB4 transcriptionally activated motifs 1 and 2, and the effect was enhanced with the 1 + 2 sequences together, indicating that motif 1 might synergize with motif 2 for transcriptional activation regulated by HOXB4 (Fig. 4c). To further confirm the transcriptional activation of DHDDS by HOXB4, we analyzed the correlation between HOXB4 and DHDDS expression in the OV samples of the TCGA database. In TCGA, OV data were downloaded and analyzed using the R package, and the results indicated that HOXB4 was positively correlated with DHDDS (R = 0.25) (Fig. 4d). However, clinical stage and grade had no significant relationship with DHDDS, suggesting that DHDDS might participate in the complex process of OV progression via downstream target genes (Fig. 4e).

### HOXB4 is dependent on DHDDS to promote OV cell progression

**Fig. 5:**
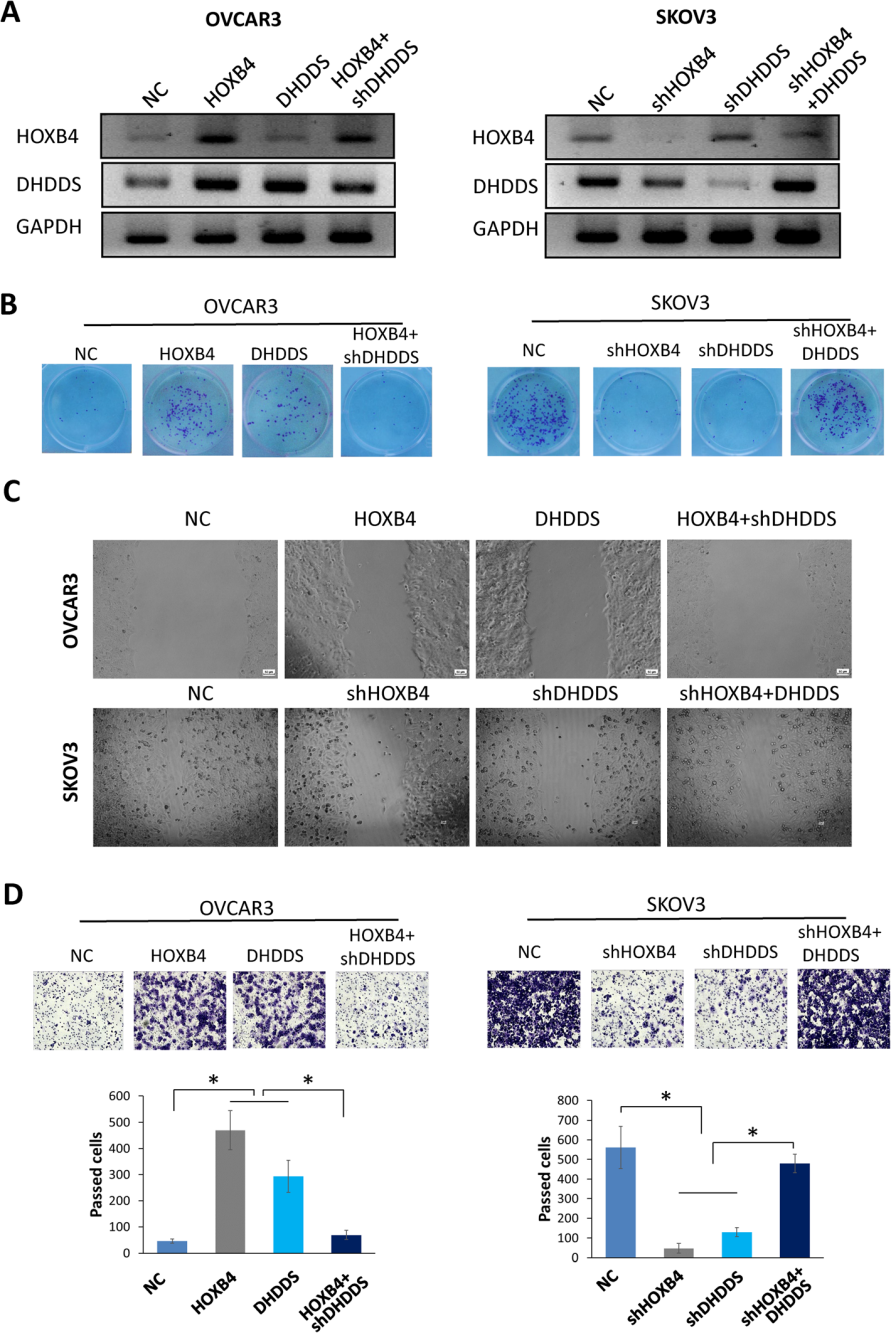
Cell function analysis of the effect of HOXB4/DHDDS in OV. **a** Western blot analysis was performed to detect the protein levels of DHDDS after treatment. **b** Colony formation assays detected that overexpression of HOXB4 and DHDDS promoted the proliferation of OVCAR3 cells, and knockdown by shRNA suppressed the proliferation of SKOV3 cells. HOXB4 or DHDDS knockdown by shRNA suppressed cell proliferation, while DHDDS overexpression in SKOV3 cells elicited neutralizing effects. **c** Wound-healing assay. **d** Invasion assay revealed results similar to those in A. HOXB4 transcriptional activation of DHDDS to promote cell progression. Statistically significant differences were analyzed using one-way ANOVA. Statistically significant differences of luciferase activity assay between two groups were analyzed using Student’s t-test, and Pearson correlation analysis was analyzed Pearson Chi square test

RNA-seq GO analysis and GSEA indicated that malignant progression, such as proliferation, migration, invasion, and EMT, may be enhanced by HOXB4 in OV. We then identified the biological function of HOXB4 in OV cells. We used OVCAR3 and SKOV3 cells to evaluate the biological function of HOXB4 in the OV process. To assess the effect of HOXB4/DHDDS on OV growth, we overexpressed HOXB4 and DHDDS in OVCAR3 cells and knocked them down via shRNA in SKOV3 cells. Western blotting was performed to detect the expression of DHDDS (Fig. 5a). In OVCAR3 cells, the colony formation results showed that HOXB4 or DHDDS overexpression promoted proliferation. Conversely, the proliferation of SKOV3 cells was inhibited by HOXB4 or DHDDS downregulation by shRNA. However, overexpression of DHDDS reversed the inhibitory effect of HOXB4 knockdown in SKOV3 cells (Fig. 5b). Wound healing assays showed that HOXB4 and DHDDS overexpression enhanced the cell migration ability. Conversely, knocking down HOXB4 or DHDDS decreased cell migration (Fig. 5c). For the invasion assay, Transwell data showed effects similar to those in the wound healing assay. HOXB4 and DHDDS promoted invasion in OV cells, while DHDDS knockdown reversed the effect of HOXB4 on OV cell invasion (Fig. 5d). These results indicated that HOXB4 exerted its effect on malignant progression dependent on DHDDS overexpression, which supported the transcriptional activation relationship between HOXB4 and DHDDS.

### HOXB4 facilitates the progression of OV in a mouse model

**Fig. 6:**
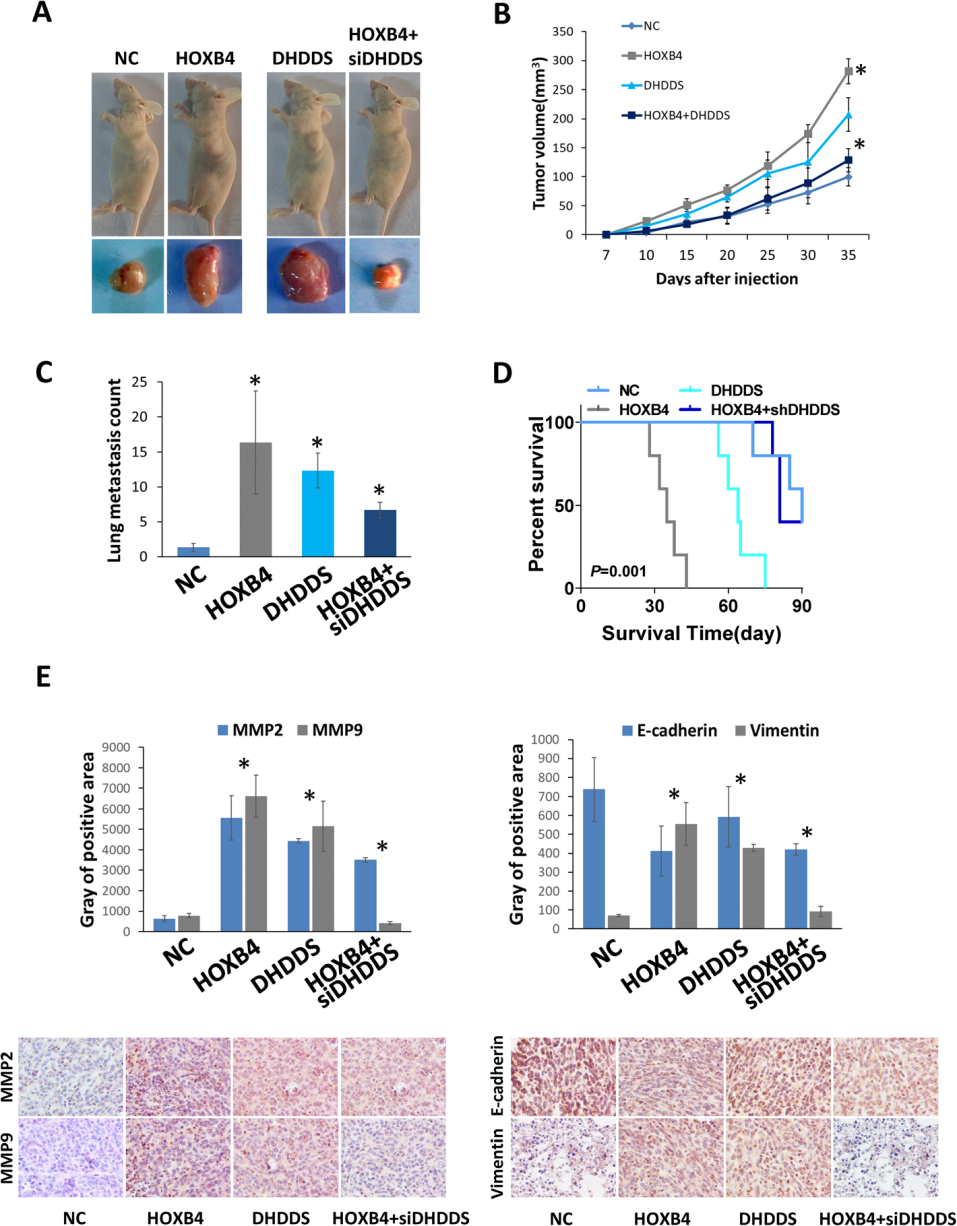
Effect of HOXB4 on the growth and metastasis of OV cells in vivo. **a** Representative images of tumors in the OVCAR3/scramble, OVCAR3/HOXB4, OVCAR3/DHDDS, and OVCAR3/HOXB4 + DHDDS-KD groups (*n* = 6 per group). **b** Tumor volume was recorded over time, and HOXB4 and DHDDS overexpression promoted tumor growth. Quantification of fluorescence in metastatic tumors (*P* < 0.01). **c** H&E staining of metastatic tumors in lung tissues and node counts; HOXB4 and DHDDS promoted lung metastasis. **d** Kaplan–Meier survival analysis of mice in different groups. **e** IHC staining of tumor samples and staining intensity analysis of the positive area to detect the progression markers for each group. Statistically significant differences were analyzed using one-way ANOVA

To further validate the role of HOXB4 in OV growth and metastasis, we established an OVCAR3 xenograft model by using BALB/c-nu/nu mice. An OVCAR3 stable transfection clone was obtained by G418 enrichment and then injected subcutaneously or in the tail veins of the mice. When HOXB4 and DHDDS were individually overexpressed, tumor growth was significantly enhanced. Conversely, when HOXB4 downstream of DHDDS was blocked, tumor growth was inhibited (Fig. 6a and b). The quantification of the number of lung metastatic nodes showed that HOXB4 and DHDDS conferred increased colonization in the lung (Fig. 6c). Survival analysis revealed that HOXB4 overexpression in OV contributed to a short survival time, and the upregulation of DHDDS also worsened the outcome (Fig. 6d). IHC staining of tumor tissues demonstrated invasion and metastasis, and EMT markers were upregulated following overexpression of HOXB4 and DHDDS (Fig. 6e). Thus, HOXB4 accelerated OV progression was dependent on its target DHDDS.

## Discussion

HOXB4 plays an important role in proliferation, metastasis, and angiogenesis in cancer [23–25, 28, 33, 35–41]. Our results indicated that HOXB4 was overexpressed in OV tissues and correlated with poor prognosis of patients with OV. The overexpression of HOXB4 promoted cell proliferation and invasion of OV both in vitro and in vivo. The transcriptional target of HOXB4 was DHDDS. Our results suggested that HOXB4 promoted the invasion and EMT of OV cells to accelerate the malignant progression of OV by upregulating DHDDS. HOXB4 overexpression has been demonstrated in OPSCC, and is associated with poor prognosis in patients. The upregulation of HOXB4 in atypical myeloproliferative neoplasms was associated with malignant cancer progression [23]. HOXB4 can modulate OV chemotherapy resistance [33]. However, how HOXB4 regulates the progression of OV is unclear. Our study confirmed that HOXB4 was associated with poor prognosis of OV and revealed that HOXB4 enhanced EMT properties in OV cells.

Previous studies indicated that HOXB4 plays a crucial role in cancer progression [24, 32, 42–45]. EMT is important during tumor metastasis in which epithelial cells lose adhesion and acquire invasive ability [46–49]. HOXB4 induced EMT and contributed to breast cancer cell migration and metastasis. Knockdown of HOXB4 inhibited EMT-related invasion in lung cancer. Our results demonstrated that HOXB4 had a substantial effect on EMT phenotypes, thus enhancing OV cell migration and invasion in OV. Although the role of HOXB4 in the progression of OV has been studied, the specific mechanism of the regulation of OV by HOXB4 remains unclear. Further investigation showed that the transcription of DHDDS was upregulated by HOXB4. This indicated that the effect of HOXB4 on OV may be mediated by DHDDS. DHDDS is involved in the biosynthesis of several types of glycoproteins in the body. Studies have shown that mutations of DHDDS cause retinitis pigmentosa [50, 51]. Although glycoproteins are crucial for tumor progression, the biological function of DHDDS in tumors has not been verified. Our results showed that the HOXB4/DHDDS axis was modulated by direct transcriptional regulation to promote invasion in OV cells. In addition, our results revealed that HOXB4 promoted OV cell metastasis and contributed to Snail and Zeb1 expression. This finding suggested that HOXB4 promoted the metastasis of OV cells by activating EMT-associated pathways.

## Conclusions

In brief, HOXB4 promoted the malignant progression of OV by regulating DHDDS. Our study verified that HOXB4 enhanced OV cell metastasis by promoting EMT and facilitated OV growth. These results implied that HOXB4 could regulate several signaling pathways, but DHDDS was the primary target activated by HOXB4 to modulate the malignant progression of OV.

## Supplementary information

**Additional file 1.**

## Data Availability

The datasets used and/or analyzed during the current study are available from the corresponding author on reasonable request.

## Abbreviations

HOXB4: Homeobox B4
OV: Ovarian cancer
TCGA: The Cancer Genome Atlas
EMT: Epithelial mesenchymal transition
PFS: Progression-Free-Survival
DHDDS: Dehydrodolichyl diphosphate synthase subunit
fetal bovine serum: FBS
GSEA: Gene Set Enrichment Analysis
TPM: Tumor mutation burden
MMP: Matrix metallopeptidase 2
