# Supplementary material for: HOXB4 promotes the malignant progression of ovarian cancer via DHDDS
Source: BMC Cancer. 2020 Mar 16;20:222. doi: 10.1186/s12885-020-06725-4 (PMC7077141; doi:10.1186/s12885-020-06725-4)
Supplement: Supplementary file 1 — Additional file 1. [file 12885_2020_6725_MOESM1_ESM.pdf]

Fig. 1B

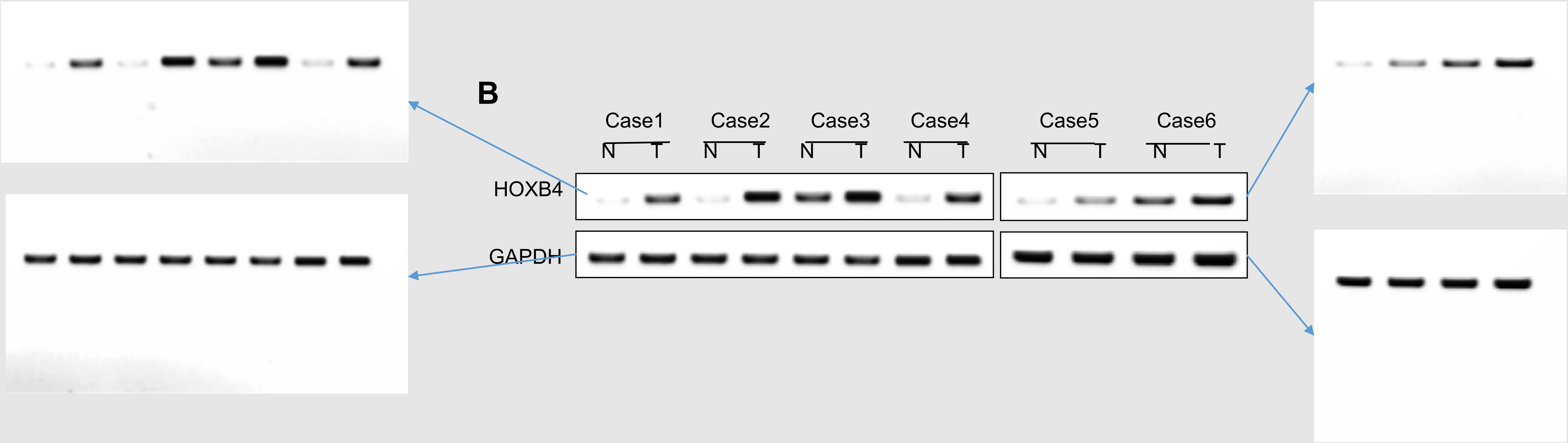
B
Case1
Case2
Case3
Case4
Case5
Case6
N T N T N T N T N T N T
HOXB4
GAPDH

Fig. 3A

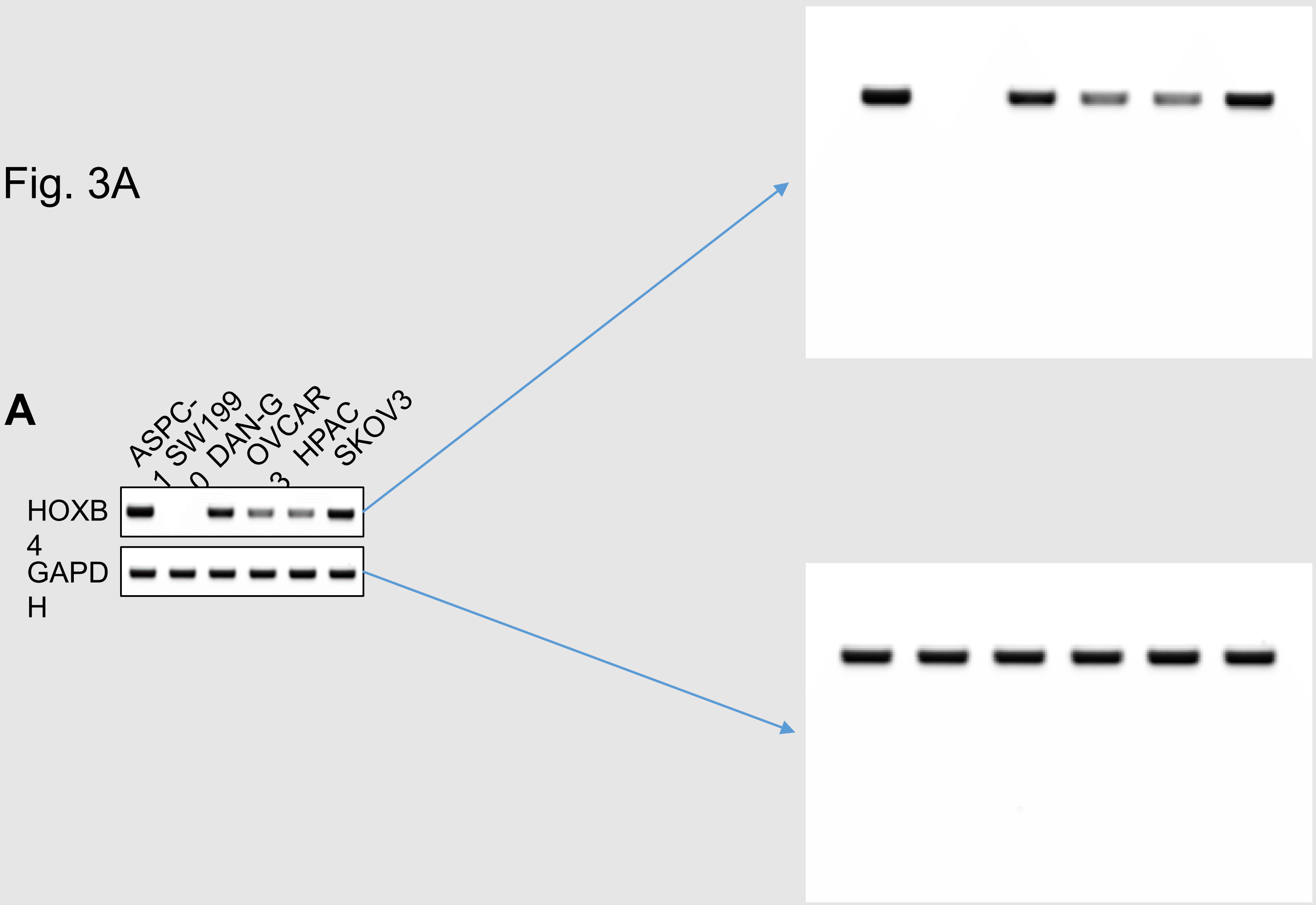

Fig. 3B

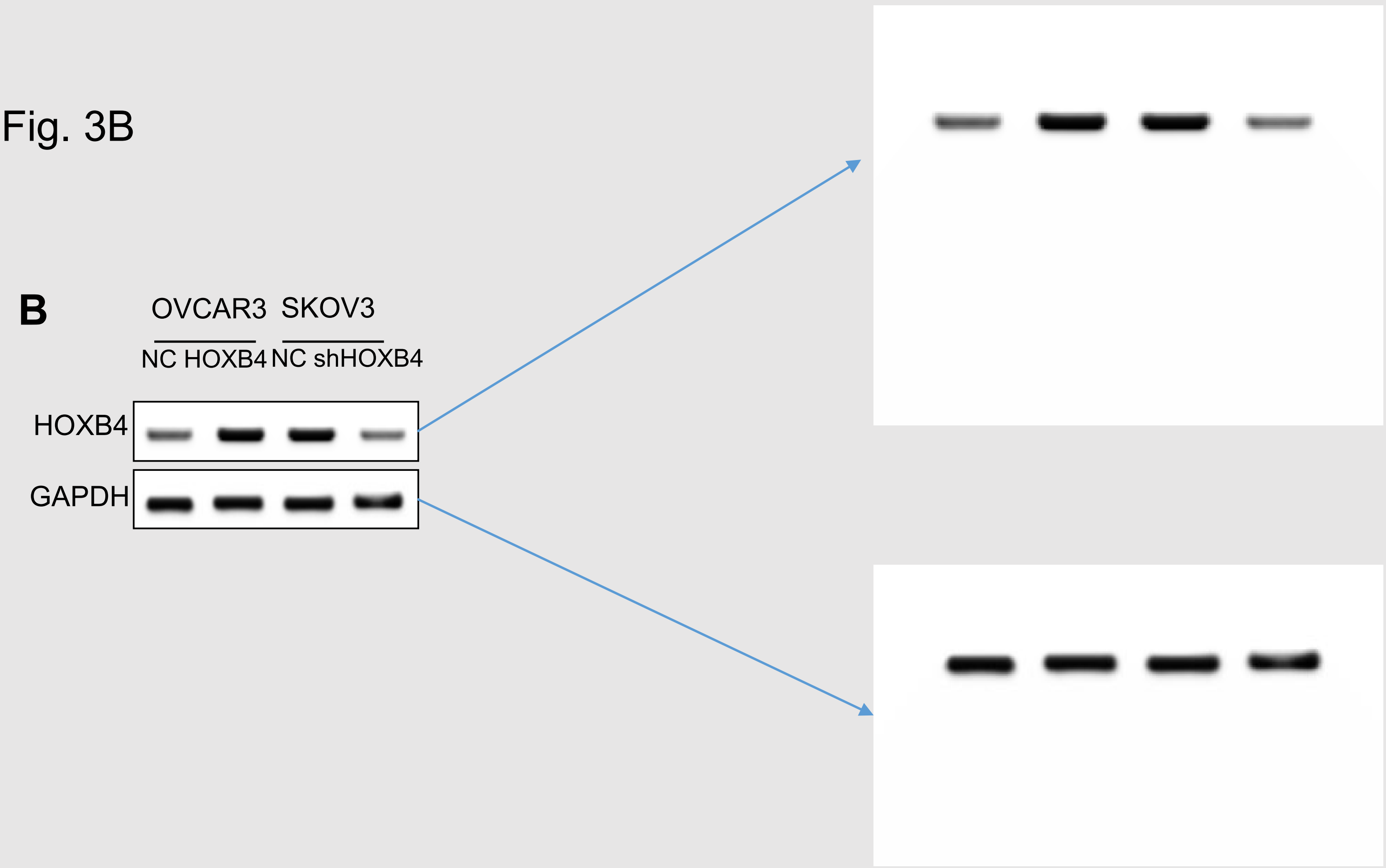

Fig. 3J

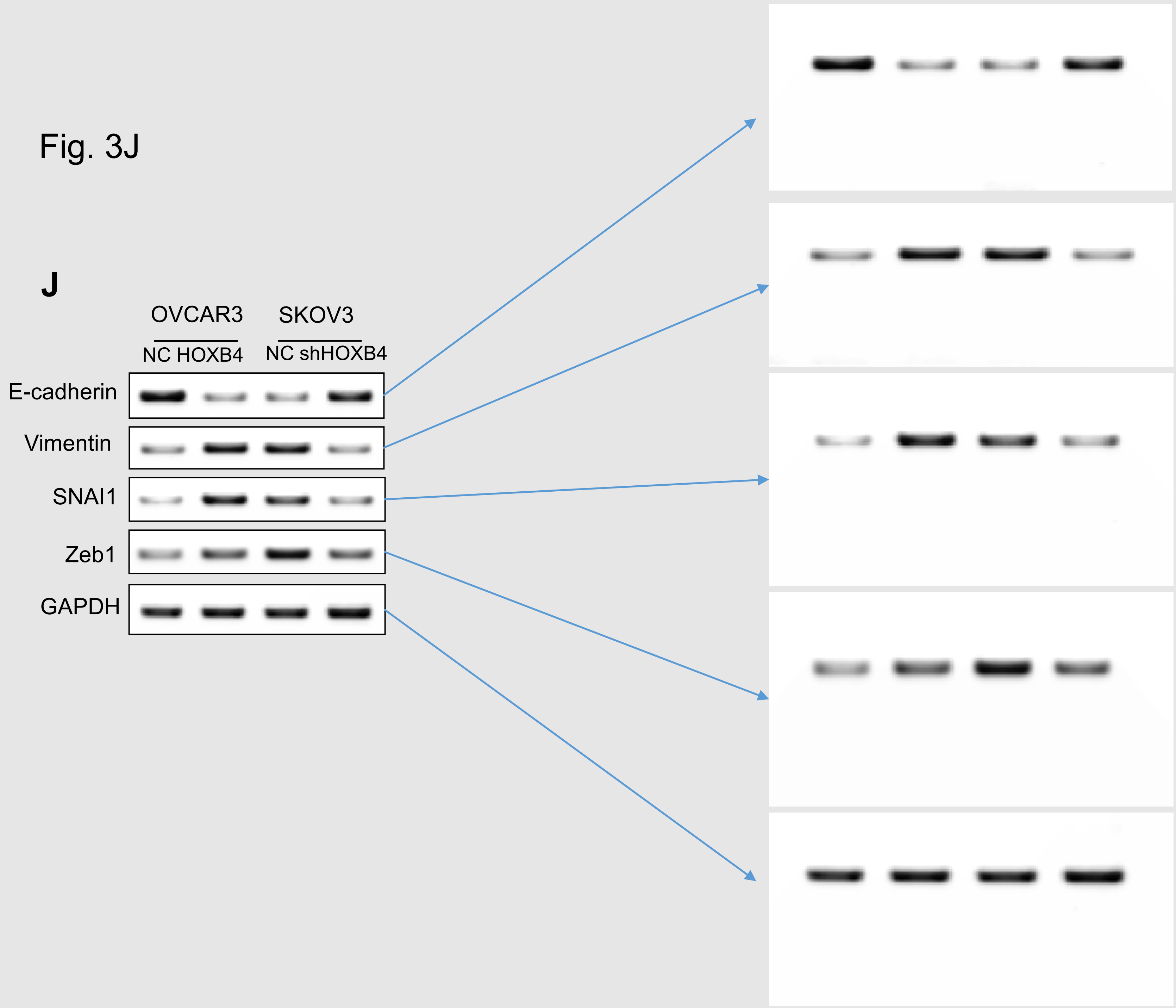
J
OVCAR3
SKOV3
NC HOXB4
NC shHOXB4
E-cadherin
Vimentin
SNAI1
Zeb1
GAPDH

Fig. 4B

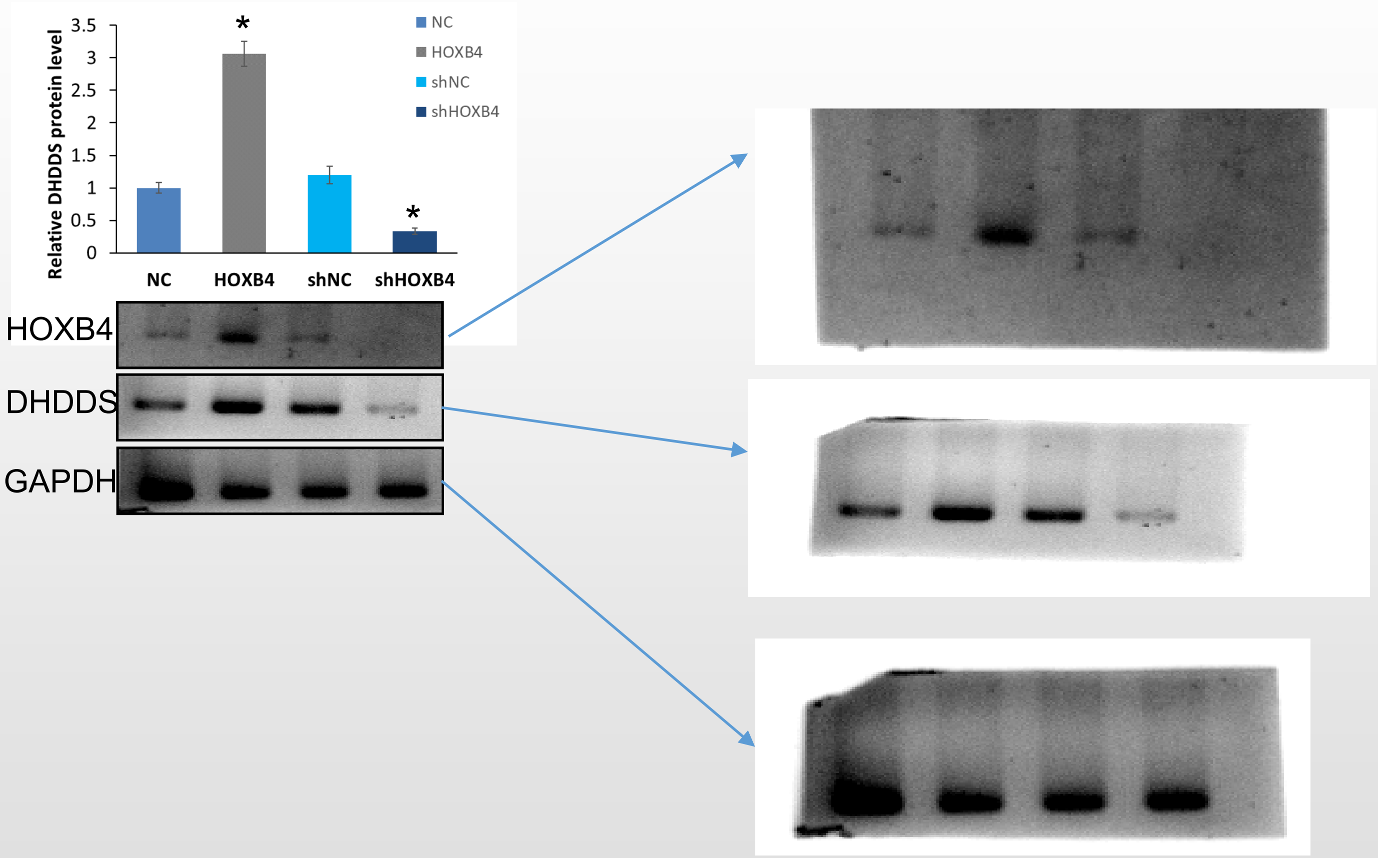
Relative DHDDS protein level
3.5
3
2.5
2
1.5
1
0.5
0
*
*
NC
HOXB4
shNC
shHOXB4
NC
HOXB4
shNC
shHOXB4
HOXB4
DHDDS
GAPDH

Fig. 5A

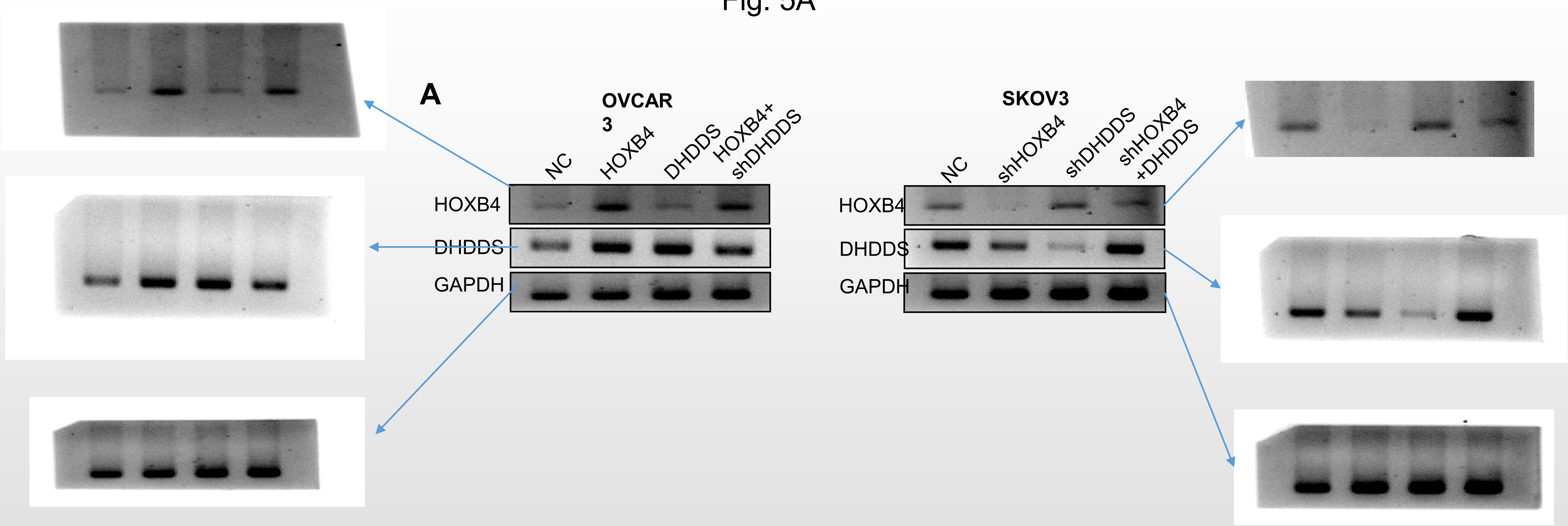

A
OVCAR 3
NC
HOXB4
DHDDS
HOXB4+ shDHDDS
HOXB4
DHDDS
GAPDH
SKOV3
NC
shHOXB4
shDHDDS
shHOXB4 +DHDDS
HOXB4
DHDDS
GAPDH
